# The meaning of nursing practice for nurses who are retired yet continue to work in a rural or remote community

**DOI:** 10.1186/s12912-021-00721-0

**Published:** 2021-11-06

**Authors:** Martha L. P. MacLeod, Lela V. Zimmer, Julie G. Kosteniuk, Kelly L. Penz, Norma J. Stewart

**Affiliations:** 1 School of Nursing, University of Northern British Columbia, 3333 University Way, Prince George, British Columbia V2N 4Z9 Canada; 2 Canadian Centre for Health and Safety in Agriculture, University of Saskatchewan, Saskatoon, SK S7N 2Z4 Canada; 3 College of Nursing, University of Saskatchewan, Saskatoon, Saskatchewan S7N 2Z4 Canada

**Keywords:** Registered nurses, Licensed practical nurses, Remote rural nursing, Retirement, Hermeneutics, Qualitative, Cross sectional survey, Canada

## Abstract

**Background:**

Although much research has focused on nurses’ retirement intentions, little is known about nurses who formally retire yet continue to practice, particularly in rural and remote settings where mobilization of all nurses is needed to assure essential health services. To optimize practice and sustain the workforce stretched thin by the COVID-19 pandemic, it is necessary to understand what it means for retired registered nurses (RNs) and licensed practical nurses (LPNs) to work after retirement. This study explored what nursing practice means for RNs and LPNs who have formally retired but continue to practice in rural and remote communities.

**Methods:**

A pan-Canadian cross-sectional survey conducted in 2014–2015 of nurses in rural and remote Canada provided data for analysis. Textual responses from 82 RNs and 19 LPNs who indicated they had retired but were occasionally employed in nursing were interpreted hermeneutically.

**Results:**

Retired nurses who continued to practice took on new challenges as well as sought opportunities to continue to learn, grow, and give back. Worklife flexibility was important, including having control over working hours. Nurses’ everyday practice was inextricably tied up with their lives in rural and remote communities, with RNs emphasizing serving their communities and LPNs appreciating community recognition and the family-like character of their work settings.

**Conclusions:**

Retired nurses who continue to work in nursing see retirement as the next phase in their profession and a vital way of engaging with their rural and remote communities. This study counters the conventional view of retaining retired nurses only to combat nursing shortages and alleviate a knowledge drain from the workplace. Rural and remote nurses who retire and continue working contribute to their workplaces and communities in important and innovative ways. They can be characterized as dedicated, independent, and resilient. Transitioning to retirement in rural and remote practice can be re-imagined in ways that involve both the community and the workplace. Supporting work flexibility for retired nurses while facilitating their practice, technological acumen, and professional development, can allow retired nurses to contribute their joy of being a nurse along with their extensive knowledge and in-depth experience of nursing and the community.

## Introduction

The COVID-19 pandemic has highlighted the challenges of nursing practice, including in rural and remote communities, where demands can quickly overwhelm available resources [1]. During the pandemic, many retired nurses have answered the call to return to nursing. Although we know that retired rural and remote nurses willingly step up during times of crisis, we know little about nurses who have formally retired yet choose to work in nursing following retirement. If we understand what these nurses consider to be the meaning of nursing practice in rural and remote communities, it may be possible to better support their important contributions to patient care and to their colleagues, both during and following health service crises, such as those precipitated by the pandemic.

Keeping older nurses working is thought to help alleviate nursing shortages [2, 3]. Research has identified the complexity of older nurses’ decisions to retire, but has overlooked the meaning of being an older nurse in general, and in rural and remote communities in particular, where the nurse’s practice is intertwined with and shaped by the community [4]. A deeper understanding of what it means to be a nurse who has retired but continues to work in rural or remote nursing can provide insights that will contribute to supporting rural and remote nurses and the communities in which they live.

## Background

There has been increased attention to demands placed on older nurses [3, 5] and the supports they require to remain in the workforce. Older nurses have been variously described as nurses who are 45 years and older [2, 6], but more commonly as those 55 years and older [3, 7]. The World Health Organization [WHO] [8] and Canadian Institute for Health Information [CIHI] [9] describe nurses 55 years and older as “approaching retirement” and “late career” nurses respectively.

Despite efforts to renew the workforce, the impending retirement of older nurses has become a concern in several countries, particularly in the American and European regions [3, 8]. This is of particular concern for rural and remote areas, which already experience shortages due to geographic maldistribution and high levels of turnover [3, 8, 10, 11]. The WHO estimates that one sixth of all nurses globally will retire in the next 10 years; 24% in the Americas [8]. The average age of nurses in Australia was 44.4 years in 2015 [12], in Canada it was 44.0 years for RNs and 41.1 years for LPNs in 2018 [11], and in the United States it was 50.9 years for RNs and 52 for LPNs in 2017 [13]. Although the numbers of older RNs and LPNs in Canada have decreased in recent years [11], 23.7% of RNs and 15.4% of LPNs are in their late career, that is 55 years and older [9].

Auerbach, Buerhaus, and Staiger [14] noted a delay in RN retirement age in the U.S., but in Canada, the shift to retirement among nurses begins at age 58 for RNs and 59 for LPNs [15]. Hewko et al. [16] found that fully 85% of RNs in the Canadian Longitudinal Study on Aging retired before age 65. These patterns in Canada have remained fairly constant [15], despite the elimination of mandatory retirement laws in Canada between 2006 and 2009. One reason for this may be Canada’s unionized nursing environment, where most nurses receive defined benefit pensions in addition to Canadian statutory pensions, which normally begin at age 65. Where nurses can financially afford to retire before age 65, it may be even more important to understand the older nursing workforce, to support late career nurses and to optimize teams within the practice environment.

Studies of nurses retiring before age 65 have identified a combination of individual, social, and work environment factors to be the main reasons for retiring early. Factors include finances, social reasons, retirement age, personal health or health of partner, a lack of challenge or development, or a lack of incentives to stay in practice [2, 3, 6, 17, 18]. Although the broader geographical context has been noted as a retirement factor [2, 15], few researchers have focused on older rural and remote nurses.

A number of studies have identified what can encourage nurses of retirement age to remain in practice for longer. Financial considerations are one such factor [6]. Three rural studies identified adequate compensation [19], increased financial remuneration [20], and financial incentives [21] as facilitators of continued employment. Workplace supports for older nurses may include ergonomic assessments or adjustments, redesigning work to lessen physical and emotional stress, control over work, and ongoing recognition for expertise [17, 18]. Supports specifically noted for older rural and remote nurses include considerations from management [21], appropriate workload, open communication, and access to professional development [20]. Opportunities for social interaction in the workplace has been mentioned as an important retention factor [18], but factors of positive social processes in the community and ensuring family and community support are only referenced in rurally-focused studies [7, 10, 22].

Studies of older RNs have mentioned the importance of flexibility in working hours and shiftwork [23]. Palumbo et al. [20] noted the importance of seasonal flexibility for rural nurses rather than only workday or shift flexibility. Flexibility also included offering rural nurses more choice about place of work over time. North et al. [24] found that New Zealand RNs, 50 years and older, left and rejoined the workforce in different practice areas, with many moving to community settings and part-time employment as they aged. Clendon and Walker [23] identified the need to better understand what nurses considered as flexibility, which could include accommodating different work trajectories for different generational cohorts [25].

Despite the need to better understand and be more responsive to different nurse types (i.e., RNs and LPNs) [23, 24], nurses are often treated as an undifferentiated group [2, 18]. Studies of workplace factors considered important to different generations of nurses have shown that professional practice environments are highly important for all generational groups [26]; however, preferences for the level and type of engagement differ. Havens et al. [25] noted that nurses over 67 years were the most engaged in the rural workplace. It is often assumed that what is important for RNs and LPNs is the same, as much of their practice overlaps. This assumption may not hold in rural and remote settings where RN and LPN practice is shaped differently by their scope of practice [27, 28] and where the determinants of their intention to leave a nursing position differs in relation to individual, workplace, and community variables [7].

In the growing body of literature on older nurses, the focus has been primarily on predicting and preventing retirement to sustain the nursing workforce. Accordingly, the majority of the research is quantitative in nature. Little is known about the challenges nurses experience when they work beyond formal retirement and what is particularly salient for them in their practice, especially in rural and remote contexts. The COVID-19 pandemic has led many retired nurses to return to practice [3]. In order for retired nurses to continue in the workforce in the post-pandemic world, where considerable nursing shortages are anticipated [3, 8], it will be important to find new ways for practitioners and policy-makers to value and support their contributions. Insights from retired nurses have the potential to provide new ways of understanding rural nursing practice and what might enhance health care continuity in these workplaces and communities.

## Methods

### Aim

The purpose of this article is to explore what nursing practice means for registered nurses (RNs) and licensed practical nurses (LPNs) who have formally retired but continue to practice in rural and remote communities. The research question was, What does it mean to be a nurse in rural and remote communities for RNs and LPNs who have formally retired but continue to practice?

### Design

This article presents the hermeneutic interpretation of textual data from a cross-sectional survey *Nursing Practice in Rural and Remote Canada II (RRNII)* [29]. The approach and methods draw in particular on the work of Gadamer [30] and Moules et al. [31] Hermeneutics is both a philosophical approach and an approach to research that seeks to examine in-depth, everyday experiences, understandings and meanings. Through a hermeneutic approach we sought to interpret what the nurses’ comments revealed about their experience, and in so doing, provide a richer and more in-depth understanding of their practice in context, than would be possible through only describing and theming nurses’ comments. Descriptive approaches such as content analysis are usually used to analyze qualitative data within surveys to depict the phenomenon of interest exactly as it is, with the expectation of direct correspondence between the words and the phenomenon’s properties and qualities [32]. Our intent was not to describe, using words from the text, but to understand meaning, which could include going beyond (or behind) what was directly said in the text [30, 33].

In this analysis, where the meaning of being a nurse in a rural or remote community is important, a hermeneutic approach drawing on philosophical hermeneutics [30] allowed us to articulate or bring into light that which was hidden or unclear. New understandings were developed through shifting our current understanding or horizon and by introducing other horizons opened by the text so we could see what the nurses say in a different way [31, 33]. Our intent was to create a new understanding, so that readers could say, “I’ve not thought of older nurses who work past retirement in this way before.”

### Participants

The survey targeted a stratified systematic sample of 10,072 rural and remote regulated nurses in Canada’s 10 provinces and all regulated nurses in the three northern territories through collaboration with provincial and territorial nursing associations and regulatory colleges. Eligible nurses practiced in a rural or remote community or were on leave for 6 months or less at the time of the survey. Rural communities were designated as those outside the commuting zones of communities with a population of 10,000 or more [34]. As there is no commonly used definition of remote in Canada, and based on findings of an earlier nation-wide survey [35, 36] where the nurses demonstrated various understandings of rural and remote, remote settings were not separately identified. We therefore refer to “rural and remote nurses.”

The *RRNII* survey was completed between April 2014 and August 2015 by Registered Nurses (RNs), Nurse Practitioners (NPs), Licensed Practical Nurses (LPNs), and Registered Psychiatric Nurses (RPNs) in rural and remote communities across Canada. A response rate of 40% was achieved with 3822 of 9622 eligible nurses from all provinces and territories; 450 were ineligible (e.g., incorrect address). RN respondents (*n* = 2082) were representative of RNs in rural and remote Canada as a whole, at a 99% confidence level with a 2.0% margin of error. LPN respondents (*n* = 1370) were representative of rural and remote LPNs in Canada as a whole, at a 99% confidence level with a 1.7% margin of error [29].

Of the 126 RNs and 42 LPNs who indicated their employment status to be retired and occasionally employed in nursing, 82 RNs and 19 LPNs provided written responses to an open-ended survey question; they are the participants for the present analysis.

### Data collection

The questionnaire was developed iteratively by a research team of 16 and advisory group of 19 nursing leaders from across the country. The comprehensive survey (27 pages in English; 31 pages in French) was available in paper or online. Survey implementation followed Dillman’s tailored design method [37] of repeated follow-up. Further information about the survey method is available [29].

The final section of the questionnaire asked an open-ended question: “What does it mean to be a nurse in rural and/or remote Canada? Please share a story, situation or experience of your everyday practice. Please give details and talk about the impact of your practice on patients/family, the primary work community and/or yourself.” All responses were in English or translated from French into English and back translated for accuracy at the time of initial entry.

### Ethical considerations

Ethical approval was received from six universities and three territorial organizations [29]. Each participant provided informed consent. All nurses were given pseudonyms.

### Data analysis

The responses were organized by nurse type (RN and LPN) and the analysis was conducted using MS-Word. Responses ranged from 10 to 4622 words, with most ranging from 50 to 180 words. Many nurses provided examples of memorable patient situations, some of which had happened over the course of long careers, while others commented about current situations. In the analysis we focused on current situations, which could be identified in the content and by nurses’ use of present verb tense.

All authors reviewed the data and the first and second authors undertook the initial, in-depth analysis and interpretation. This process included MM and LZ independently reading all of the responses several times, identifying what stood out and then continually comparing and contrasting nurses’ comments. Each of these authors created an initial organization of the findings, which they discussed and together, determined a working organization of findings. Hermeneutic research is about creating a coherence of the whole and its details, while embracing plurality and difference [30, 31]. We sought consistency between the philosophical assumptions, the conduct of the analysis, and the expression of the conclusions [31].

The authors engaged in dialogue and writing/rewriting to further shape the analysis. The hermeneutic circle [30] was evident throughout the iterative process, in which we identified salient factors with illustrative quotes, that were recast when seen in the context of the whole. This then led to consideration of the factors, again leading to a refinement of the whole. The results of the hermeneutic interpretation are expressed as interpretive findings of sets of comments [31].

### Rigour

Consistent with a hermeneutic approach to research [31], we did not try to remain un-biased. Three of the authors are over the age of 55; two are retired RNs. We actively kept our pre-understandings in question, both individually and through the course of our dialogues. Consistent with hermeneutic approaches to rigour [33], we kept notes of analytic decisions. In analyzing the data, we attempted to ensure that the selected responses fully reflected credible, meaningful interpretations, which would be useful both practically and theoretically in other contexts [33]. We aimed to create a plausible account that was trustworthy and reflected the data [33]. That is, the account would fully resonate and be useful in extending the readers’ understanding.

## Findings

### The nurses

Table 1 outlines the characteristics of the 82 RN and 19 LPN participants. The RNs ranged in age from 57 to 82 years, with a mean age of 64 years; LPNs ranged in age from 51 to 69 years, with a mean age of 61 years. The majority of RNs (58.7%) and LPNs (82.4%) were aged 64 and under. One RN was male, all others were female. Ninety percent of RNs (90.2%) and 78.9% of LPNs worked less than full-time, most often on days. Most RNs (82.5%) and LPNs (94.1%) worked as staff nurses, with RNs working in a wider variety of settings than LPNs, who worked primarily in hospitals (52.6%) and long-term care (31.6%). Most RNs (63.7%) and LPNs (78.9%) had been employed by their primary employer for 20 years or more. This would indicate that many nurses were long-term residents of their communities or regions.

**Table 1:** Characteristics of nurses who are retired and occasionally working in nursing, RNs (*n* = 82) and LPNs (*n* = 19)

	RN^a^ n (%)	LPN^b^ n (%)
**Gender**		
Female	73 (98.6)	18 (100.0)
Male	1 (1.4)	0 (0)
**Age**		
45–54	0 (0)	2 (11.8)
55–64	44 (58.7)	12 (70.6)
> 64	31 (41.3)	3 (17.6)
**Highest attained nursing credential**		
Diploma	56 (69.1)	19 (100.0)
Bachelor’s	22 (27.2)	0 (0)
Master’s or Doctorate	3 (3.7)	0 (0)
**Hours worked in nursing**		
Less than full-time	74 (90.2)	15 (78.9)
Full-time	6 (7.3)	4 (21.1)
More than full-time	2 (2.5)	0 (0)
**Shift worked most often**		
Days	61 (74.4)	11 (57.9)
Evenings	5 (6.1)	3 (15.8)
Nights	8 (9.8)	2 (10.5)
Rotating	8 (9.7)	3 (15.8)
**Current primary position**		
Manager	5 (6.3)	0 (0.0)
Staff nurse	66 (82.5)	16 (94.1)
Nurse practitioner, Clinical nurse specialist, Educator/researcher/consultant/analyst	9 (11.2)	1 (5.9)
**Primary place of employment**		
Hospital	36 (44.4)	10 (52.6)
Community-based health care	23 (28.4)	1 (5.3)
Nursing home/Long-term care	14 (17.3)	6 (31.6)
Private nursing, self-employed, educational institution, government, professional association	8 (9.9)	2 (10.5)
**Population of primary work community**		
999 or less	20 (25.3)	4 (22.2)
1000–9999	41 (51.9)	13 (72.2)
10,000 or over	18 (22.8)	1 (5.6)
**Duration of employment by primary employer**		
> 10 years	19 (23.8)	4 (21.1)
10–19 years	10 (12.5)	0 (0.0)
≥ 20 years	51 (63.7)	15 (78.9)

^a^ Sample size varies from 74 to 82 due to missing responses

^b^ Sample size varies from 17 to 19 due to missing responses

### Interpretive findings

#### “I can’t bring myself to give it all up entirely”: retirement as a phase in a nursing career

The nurses who were retired yet continued to work in rural and remote communities, as one might expect, expressed a wide range of comments. The first set of comments related to what it meant to themselves and their communities to be in the situation where they had already retired, yet continued to practice. The second set of comments concerned how the nurses made it possible for themselves to continue working following retirement.

#### Being an older, retired, still practicing nurse in a rural and remote community

When the RNs and LPNs wrote about what being a nurse meant to themselves and their communities, they commented on the challenges of coming to retirement, what they loved about nursing, and what rural and remote nursing allowed at this stage of their careers. It included giving back to their colleagues, giving to their community. In turn, they received trust, respect, and gratitude from their patients, families, and communities.

#### “When I really retire”: the challenge of coming to full retirement

In answering a general question about what it means to be a rural nurse, it was remarkable how many wrote directly about being retired yet continuing to work in nursing. It suggested that continuing to nurse while being retired was important to the nurses themselves. Many referred to nursing as a central part of their identity, their calling. “First and foremost, I am a nurse, always have been – always will be” (Margaret, RN). Nurses wrote about the pleasures of being a nurse, what it means for them, and how they structured their work-lives following retirement:

I have been Nursing for almost 45 years. Retired 7 years ago [at age 59], still do casual. Enjoyed nursing all of my life. Yes there are challenges, but the good outweighs the bad. I consider my nursing career as my "calling" in life, I love helping people and being there for them. … Have paid my license [for two more years], will decide then about continuing. I work casual so I don't do 12 hours shifts, do some 8 hours- 4 hours- cover off busy times etc. (Emma, RN)

Emma was not alone in experiencing the difficulty of making the decision “to completely retire” (Lili, RN) or “really retire” (Susan, RN). Others noted that “one can’t work forever” (Nora, RN), that things change and it is sometimes difficult to keep up and to deal with the workload:

I am getting ready to retire. I know I am stressed out, burned out and just plain tired out. But I can't quite bring myself to give it all up entirely. I know now I have to transition myself away from nursing, as I know my limits. Nursing is fast paced and technologically challenging at times - even in remote and rural settings. It's getting harder to keep up with continuing education and maintaining a “normal home life.” (Margaret, RN)

In addition to the mental/emotional stressors, a few nurses wrote about the physical toll of nursing, for example, that heavy lifting, “tends to wear on bodies through the years” (Ada, LPN). Several spoke about not doing 12 h shifts any more, alluding to the stress of working longer shifts. Others noted that high turnover in rural facilities with a lack of replacement staff lessened their willingness to continue to work, “it’s a dire situation. The remaining nurses are working alone and covering shifts. Very scary!” (Sandra, RN). Healthcare delivery reorganization and related demands such as, “travel, politics, and too large an organization which always created challenges in doing the prevention/promotion work effectively” (Debbie, RN), along with increases in “paperwork” (Sara, RN) and workplace changes made worklife less appealing:

I feel no connection to my work as I did before and the passion for my work has been crushed from above. My supervisor, she is good but driven from above. As well, we are not allowed to make any decisions on our own anymore. Everything must be the same in each community. (Linda, RN)

These personal and workplace factors contributed to nurses’ initial retirement. They were offset in part however, by opportunities to work after retirement in ways that allowed the nurses to continue to work in nursing, with different sources of satisfaction.

#### “And still learning”: loving the autonomy and challenges

One of the LPNs and many of the RNs talked about the pleasure of working in small communities, both because of the challenges and despite the challenges, “The pace is quick and constant learning, occasionally stressful” (Ann, RN). It seems that there was a balance of maintaining confidence in one’s knowledge and skills, while learning more:

My recent return to occasional work in a long term care facility in a small rural setting has been somewhat nerve-wracking and sometimes overwhelming because I never worked in any other setting [than a mental health community centre] let alone one requiring the application of basic nursing skills and being placed in a supervisory position on evenings and weekends. The whole experience for me is best described as very challenging, especially with no access to nursing colleagues during one's shift. I especially feel confident and in control when working near to or with the family members of a patient in the palliative care phase: because of my knowledge and comfort with assisting people in crisis, in grieving and in understanding their many needs and difficulties during this time. (Sharon, RN)

The ability to move into different areas of nursing did not stop at retirement, nor did learning within familiar environments. Many RNs wrote about continuing to learn in retirement. “There is something new to learn every day… I still enjoy the challenge of the complicated patient and like to support the recently graduated nurses as they begin their career as rural nurses” (Patricia, RN) and “nursing in a rural area constantly challenges one’s skills/knowledge” (Nancy, RN). In small communities, nurses needed high levels of knowledge not only during their working hours, but also to maintain community trust outside of work:

Being known in a community as a nurse often means many phone calls at home for advice or reassurance. In order to maintain competency and the "good will" of the community means many trips to the city for conferences, workshops, learning opportunities. With the lack of physicians in our area, more and more is being asked and expected of nurses, both in patient care and patient assessment. (Chris, RN)

As nurses in rural and remote areas “are generalists, specializing in all aspects of nursing care with very little back-up” (Nancy, RN), nurses were required to gain and maintain a breadth of knowledge and skills throughout their career. As one RN wrote, “We do it all which keeps us sharp and skilled in all areas” (Lillian, RN). Others worked part time as a way to keep engaged and up to date, “[I] am still learning. I still do a few casual shifts as needed and keep involved in any ongoing [sessions] and workshops to keep up to date” (Ann, RN).

#### Giving back: being a mentor and teacher

A few RNs spoke about being a mentor and teacher of students and new nurses along with the satisfaction it brought them. One RN communicated it particularly well:

Working in a rural nursing setting allows you to use all your skills and problem solving capabilities. It provides opportunities to be independent and innovative. In my current role, I mentor new graduate nurses to assist them in working in a rural setting. We provide supports and experienced nurses to work with new graduates to enable them to fulfill their nursing role in some fairly isolated settings. In order to ensure adequate nursing staffing levels in rural facilities, these mentoring roles are essential. (Mary, RN)

For some, mentoring was the pleasure at the end of a career:

I practised as a nurse for 25 years, which I loved. In the more recent years, I have been teaching and working as a mentor; what a wonderful job. I am still working (after 30 years of retirement) in a laboratory to take blood samples in the mornings (shortage of staff). This year I will retire for good. (Lise, RN)

The notion of giving back near the end of a fulfilling career was important in many nurses’ professional lives.

#### Belonging in the community: contributing to the community

The nurses who had retired and continued to work provided a glimpse into what it means to be an older nurse in a rural and remote community. They had a wealth of generalist knowledge and skills along with nuanced knowledge of the community, its people and resources. For some, this was derived over years as neighbors and community members, as well as professionally as nurses. This knowledge of nursing and the community allowed nurses to give family-centred care in innovative ways. A home care nurse gave an example:

Nursing in a rural area constantly challenges one's skills/knowledge…. As an RN in the Direct Service Nursing area of the Home Care Program, I faced a situation today [Friday] with one of my clients. This person is an 83 year old diabetic with a chronic ulcer on his heel. He lives with his 80 year old wife in their own 2-story house. He would be incapable of living on his own if his wife was unable to provide much care for him. Today his wife (who is not my client) reported that she was unwell, BP 80/60 (takes by herself), "dizzy,” and diarrhea (6-7 times in 4 hours). She had undergone a colonoscopy yesterday. My dilemma was how to care for my client, knowing that his caregiver was not well, knowing that my supervisor only works two days a week, knowing that our facility was not on-call over the weekend. My solution was to strongly encourage the wife to telephone the clinic and/or lab to have blood work done. I followed that up with a phone call to the clinic to ascertain whether they had talked to her. Not taking this step may have resulted in an EMS call to have both husband and wife admitted to a facility for care. Incidents such as this make rural nursing interesting and challenging. (Nancy, RN)

This RN acted quickly on a Friday, knowing the resources in the community and that there was no one else to take the step of caring for her client’s caregiver. She acted with confidence and did not draw the line at only narrowly caring for “my client.” Nancy drew on her extensive skills, even after many years in practice, which not only made it interesting for her practice, but also served the patient, family, and community. It allowed her, as another RN said, “to use all your skills and problem solving capabilities. It provides opportunities to be independent and innovative” (Mary, RN).

Retired RNs frequently expressed pride in belonging to the community and a commitment to it. They talked about having “a positive impact on our community” (Judy, RN) and contributing to the workplace when short staffed. RNs consistently referred to serving the community as an honour:

[I am] extremely proud of the work I have done [over 40 years] and of the commitment I have made to my community and the people my work community serves. It has been my honour to serve several generations of patients/clients and their families in my practice. (Barbara, RN)

That honour was reciprocated in many cases by gratitude from community members. Gratitude was shown through trust and thanks, “To receive thanks in public places as wells as the hospital or clinic is very gratifying” (Chris, RN), and expressions of safety, “the whole population knows me and they appreciate me very much … my patients… say that they feel safe with me because they have known me for two generations” (Cecile, RN).

RNs noted that personal relationships with their patients shaped their practice to be more patient-centred. It often meant “dealing with the whole family” (Marianne, RN), with the “self-satisfaction of dealing with clients/family/friends that know and trust you.” (Maria, RN):

I have worked in the city but find the rural setting more personal, knowing most of the patients and families. It also helps the residents, as I remember them when they lived in the community and before they had dementia. In the city I never had the same connection with the patients/residents, although if they stayed in the hospital long enough you would get to know them. (Sara, RN)

Retired LPNs also indicated how working in the rural community gave them satisfaction, especially the connection with patients and families. While LPNs did not talk about serving the community, they related the satisfaction of living and working in a small community, “… as being in a smaller community, you are able to work with or nurse people you have known all your life” (Laura, LPN). As well, in the small communities, “People come up to you to thank you for the care you gave them over the years” (Connie, LPN) and “people stop you on the street to say they have not seen me at work and they miss me!” (Ada, LPN).

Many LPNs commented how working in a rural community felt like working in a family:

Nursing in a rural community is like family. We share stories together and we relate to each other about happy or sad events. We sometimes socialize together, which is a stress reliever. Although I am retired I feel a sense of belonging. (Marta, LPN)

An RN working in mental health wrote, “Being a smaller facility - census usually around 25 - we find a good relationship between staff. Our approach to nursing care is always a team approach - Physicians, nurses, social workers, psychologist, unit coordinator” (Donna, RN) and another mentioned, “It’s a pleasure to go to work when you are welcomed enthusiastically by the whole team you work with” (Cecile, RN). While the LPNs described work environments as family-like, both RNs and LPNs commented on appreciating close working relationships in the small facilities, while acknowledging that workgroups were not always stress-free:

Rural nursing is usually very satisfying. You most often know the patient or family…. The staff are like family (most of the time), they are willing to trade shifts as needed and our coordinator is very understanding. Rural nursing is usually family oriented…. There is no perfect place to work. You will always have at least one pot stirrer or bully on site. Mostly if you work together as a team you can manage to have a little fun and get the job done. (Julia, LPN)

#### Making it possible to continue working following retirement

In the second major interpretive finding RNs and LPNs described how they made it possible for themselves to continue working following retirement. They accomplished this primarily through ensuring flexibility and control in their work commitments and exploring new and evolving roles. The nurses expressed the freedom to continue fulfilling their nursing identities while taking advantage of the benefits of retirement.

#### Having flexibility and control: “I work my own hours”

Many RNs and LPNs mentioned that they moved to part-time or casual work after retirement, which came with personal and professional benefits. Several identified the importance of the flexibility and control offered by casual work. It meant choosing their shifts, choosing locations, taking up new roles, and expanding knowledge.

Working as a casual nurse allowed the nurses to choose their shifts: “I work my own hours and happy with that” (Chris, RN). Some of the nurses took a different approach to flexibility by working in blocks of time, such as a nurse who had been retired for 7 years:

I am working casual 40-50 shifts in a 6 month period (some 4hrs, 8 hrs and 12 hrs shifts) picking and choosing mostly when I want to work. The other six months is spent wintering in Florida - the best of both worlds. (Wendy, RN)

Both RNs and LPNs reported the benefits of working on a casual basis, including the opportunity to contribute, to learn, to be challenged, and to keep engaged with colleagues and nursing practice. As one LPN wrote, “Working casual allows me to keep in touch with the patients, keep my nursing skills, and stay in touch with my colleagues” (Kathy, LPN), while another noted how working casual contributed to the workplace, “I feel I can help with staffing - Give good care” (Carol, LPN).

Freedom was also offered by retirement, in terms of financial security and fulfilment through work. As one RN noted, “Retirement wages ‘don’t cut it.’ So financially, this opportunity has enabled me to do things I would never have been able to achieve…” (Jean, RN). Another RN who started her own business wrote, “At this point in my life I enjoy the flexibility of the job and get to enjoy nursing in the role I am most comfortable” (Charlotte, RN).

#### Evolving roles: taking advantage of new opportunities

Following retirement, many RNs and almost all LPNs reported moving to part-time or casual work in their own organizations, including moving from acute wards to those less acute-focused. Others took on new nursing roles or similar roles in new situations. Several nurses relayed that they formed their own businesses and several RNs identified how they took up fly-in, fly-out positions in remote communities.

The newly-created businesses drew on the nurses’ previous experience and roles. An LPN wrote about doing pre-employment histories and drug and alcohol testing for local companies, working out of home by appointment (Elaine, LPN). An RN who had been a manager before retirement, “created a business proposal” for a business doing “nursing assessments for two agencies” that provide home care services (Charlotte, RN). Another LPN created a business in providing “private footcare” (Nellie, LPN).

Other nurses took up opportunities in new clinical areas, moving from public health to a shift manager role in a mental health hospital, to work in remote communities, “I work 3-4 communities per year” (Joyce, RN), or to work differently in familiar communities. “I …work casual 5-6 weeks a couple of times a year. I frequently return to the community where I worked prior to my retirement. I also work in a variety of other communities in the [territory]” (Therese, RN).

RNs talked of drawing on skills learned throughout their careers that made it possible to take advantage of these opportunities. Others commented, “My nursing career has afforded me many skills that are transferrable” (Dora, RN) or noted taking on roles in new clinical settings. An RN, who is working for the first time in a small hospital, wrote:

My past 40+ years of experience as a general duty nurse and a nurse manager has given me the tools to provide the essential services required and opportunity to work in many different areas. I also have been … teaching students in the clinical setting. (Maria, RN)

Many of the retired nurses drew on their years of experience to work in new, complex situations with varied demands. As one RN wrote:

It is a brilliant career with extraordinary people in the community. In 35 years I have always loved my work in nursing and it is quite varied, however, this [public health job] has helped me grow the most… Independent, rural and remote practice is the place to be to use all that a nurse knows. (Janet, RN)

The opportunities included working part time, working differently than before, and in different settings or roles. A notable thread that ran throughout many of the RNs’ comments was the pleasure of the variety in rural nursing and the challenges it brings.

## Discussion

When nurses who are retired and continue to work write, “I can’t bring myself to give it all up entirely,” they are speaking of their work with patients and families, the roles they play in their workplaces, as well as their professional identity in themselves and in their community. The difficulty for nurses in completely retiring is tied up with their commitment to their profession, themselves, their patients, and communities. They cannot leave nursing behind because nursing is a large part of who they are.

The nurses who wrote about planning to retire referred to several workplace and personal issues raised in studies of intention to leave practice or to retire [2, 7, 38]. Both Markowski et al. [2] and Fisher et al. [39] identified factors that push towards retirement or pull towards continuing to work. Among the main personal push factors were health and age [2], while job satisfaction and organizational commitment were important pull factors [2, 7]. The professional value of being a nurse was identified as a pull factor as well [2, 23].

What sets these rural and remote nurses’ experiences apart is how their everyday practice is played out in rural and remote communities. Small communities are individually shaped by the geography, people, economic base, and available services, where place and situation bear a much greater influence on nurses’ experiences of their occupation and their lives, than in urban settings, where the workplace itself figures more strongly in older nurses’ experience [5, 10]. Embedded in their communities, retired nurses, with their knowledge of the community’s people, services, economy, and geography, as well as their often longstanding network of relationships, provide many important links between community and workplace that contribute to both patient care and the healthcare team [40].

Missing from the nurses’ comments were some of the challenges of aging identified by rural Australian nurses and other health workers, which included the uniquely rural components of meeting the demands of driving long distances including at night, and hearing speech in video and teleconferencing meetings [41]. Although these indeed may be issues, they did not figure into nurses reflecting upon what it means to be a rural or remote nurse. Except for one retired RN’s comment, the importance of finances was also notable by its absence. While financial remuneration may be important to older nurses [2], it was not top of mind when the nurses reflected on what it meant to be a rural or remote nurse.

While in many ways, RNs and LPNs had similar comments about what it meant to be a retired nurse in rural and remote communities, there were notable differences. LPNs commented on the importance of feeling part of a family in both the community and the workplace. As well, their comments about the importance of collegiality and the pleasures of being acknowledged by the community are consistent with Gan’s [18] findings about the importance of social connections to older nurses’ continuing to work. Although RNs also commented on social connections, their consistent emphasis about serving their patients and their communities stands apart from current research on retention of older nurses. Both RNs and LPNs noted the pleasure in hearing gratitude for their work from community members. It provided a means of satisfaction and affirmation. It also was a direct indication that they were doing a good job as nurses. As Day et al. [42] note, gratitude is important in enhancing nurses’ worklife and caregiving relationships. For retired nurses in rural and remote communities, gratitude is as important to have from the community as it is from the workplace. For RNs, the gratitude from community members reflects and reinforces their professional commitment and commitment to serve their community.

The differences expressed by retired RNs and LPNs may relate in part to the setting and scope of their practice. MacLeod et al. [27, 28] detailed the differences in the scope of practice of RNs and LPNs, as did Stewart et al. [7] about differences in RNs’ and LPNs’ intention to leave their practice. The RNs’ broader scope of practice sets up different opportunities for new nursing roles in retirement as well as for professional development.

Common among nurses’ comments were the pleasures and opportunities afforded by continuing their career after retirement. Retirement underscored the resilience of nurses who viewed this stage not as a setback but an opportunity for new roles, to learn and be challenged, and to engage in workplaces and communities in new ways. Both RNs and LPNs established new businesses, bringing their long experience to bear in practice with patients, organization, and management. Many RNs talked about relishing the challenges and opportunities to keep learning and developing in practice by taking on new roles, working in new settings, or continuing in the rural and remote setting where practice challenges are many and varied. While LPNs did not express as many statements about learning and developing, they did comment on keeping current. The retired nurses’ tone of optimism and a view to the future contrasts with an ageist perception of decreased physical and mental capacity of older workers [43] and the contention that older, “veteran” nurses often seek to avoid, block, or slow changes in the rural or remote workplace [40]. The retired nurses’ positive and resilient outlook raises the possibility of re-conceptualizing how new technology and care modalities are implemented. Fruitful approaches may build on older and retired nurses’ commitment to nursing, their patients, and their community, as well as on their desires to remain engaged, be challenged, and learn. Albeit some retired nurses noted the demands of new technology, the enthusiasm and commitment of others raise new possibilities for nursing teams from several generations to continue to learn together, and help each other learn new technology to provide improved care for patients and their communities.

The majority of retired RNs and LPNs worked part-time and/or in casual roles, and noted the importance of flexibility and their ability to control when and how they work. Their comments echoed Clendon and Walker’s [23] identification of flexibility in many forms, including flexibility in hours, weeks, months, location, worksites, and careers. Flexibility and control took many different forms in the retired nurses’ comments, with LPNs expressing fewer types of flexibility than did RNs. Nurses’ control over their work lives was directly related to the flexibility both retirement and work opportunities afforded them. The comfort of retired nurses with continuing to work when they have control is hinted at in recommendations for policies that support flexible approaches to staffing [8, 10, 44]. The retired nurses’ comments suggest, however, that policies and approaches would be most successful if they empowered managers and staff in small, rural facilities to be both flexible and fair in addressing the needs and realities of the team in the context of their community.

Many of the retired nurses who continued to work in rural and remote communities changed their work to suit their changing circumstances, which is reflective of the notion of retirement transition. Bordia et al. [45] note the complexity of transitioning to retirement and conclude that pre-retirement role identities that can be continued or adapted during and after the transition period are most helpful in making the move from work to retirement. The retired nurses’ strong focus on their professional identity that is reinforced within their multiple personal networks in rural and remote communities suggests that innovative approaches to retirement transition are possible in these contexts, where the community and workplace are intertwined.

Beehr and Bennett [46] and Fisher et al. [39] discuss the role of bridge employment, that is paid work after retirement and before complete labour force withdrawal, in making the transition to full retirement. Employment during the COVID pandemic in contact tracing and vaccination clinics may have served as a type of bridge employment for retired nurses, offering control over work hours while providing new challenges. What is yet to be known is how pandemic-related employment and continuing professional commitment will influence retired nurses and their practice once the pandemic is over.

In conjunction with insights arising from the pandemic experience, it would be useful to consider the skills, needs, and satisfiers relayed by the nurses that could characterize bridge employment opportunities in rural and remote settings. It may be fruitful to widen considerations for bridge employment to include opportunities within the evolving, integrated primary and community care sector that could be taken up by entrepreneurial retired nurses. Such opportunities for retired nurses throughout the health system may foster a more welcoming place for younger nurses to begin their careers, while valuing and honouring the wealth of nursing and community knowledge that retired nurses hold.

The retired nurses’ comments echo much of the literature about retaining and supporting older nurses. When nurses retire and leave rural and remote practice settings, their complex generalist knowledge of nursing practice and their communities is lost. Newer nurses are impacted directly, with fewer nurses available to provide care, and indirectly, by the absence of crucial knowledge that older nurses pass on to those entering practice. Even though mentorship roles have been identified as a retention tool for older nurses [2, 18, 21]. it is not enough to focus only on how older nurses can be used. The nurses’ comments show the importance of valuing their knowledge and practice, with an end-in-view of enhancing rural and remote practice itself. Policy initiatives and workplace practices that pay specific attention to the contributions of nurses who have retired and continue to work in nursing are needed. Efforts will be more successful in enhancing nursing practice and the care of communities, when such initiatives and practice are place-based and nuanced in ways that suit the needs and realities of the nurses, practice settings, and communities themselves.

Our previous regression analysis of determinants of intent to leave a nursing position in rural or remote Canada [7] provided a population-based perspective from regulated nurses prior to retirement. This hermeneutic analysis of experiences of a subset of pan-Canadian RNs and LPNs who stay in nursing after formal retirement adds new knowledge from the reported actions of nurses as opposed to reports of intentions. These complementary approaches provide a strong knowledge base to inform health services policy and future research on rural and remote nurses on a global level.

### Strengths and limitations

A strength of this study is that it captures the thoughts and experiences of rural and remote RNs and LPNs across Canada. Consistent with hermeneutics, this analysis offers only one perspective of a phenomenon that could have been viewed differently [30]. The interpretation of the retired nurses’ comments offers a glimpse of what it means to be retired and continuing to work as a nurse in rural and remote communities. Further research with rural and remote nurses approaching and entering retirement is merited to explore other issues, such as what happens to nurses’ identity within rural and remote communities and how that identity figures into returning to work after retirement, as well as lessons learned from the innovative ways this group approaches retirement.

## Conclusion

“I can’t bring myself to give it all up entirely” is a metaphor for one common denominator of what it means to be a nurse in rural and remote communities for RNs and LPNs who have formally retired and are continuing to practice. Many of the nurses are working because they love it and cannot leave it behind. Nursing is who they are. That, in and of itself, can set a tone in the workplace. Through having some control over when and how they work, the nurses have been able to let go of the more troubling aspects of nursing employment and enjoy nursing practice. There is a sense that the nurses continue to work following retirement for their patients, for the people of their community, and for the joy of serving, along with the satisfaction that comes from it. Although both RNs and LPNs express a depth of commitment and love for the profession, they express it differently, with a different emphasis on professional commitment and its possibilities. The nurses’ engagement in nursing and their communities sets up the conditions for them to continue to learn and respond to challenges in everyday practice. Through that engagement, they help others provide an essential continuity of care.

The expressions of the retired nurses about working and living in rural and remote communities raise several considerations for future action in clinical practice, as well as in supporting a multigenerational workforce. It is not enough to focus on retaining older nurses through improving working conditions. Nor is it enough to consider the older nursing workforce only in terms of how to extend their work lives so as to stave off nursing shortages, such as those exacerbated by the COVID-19 pandemic. By reconsidering what nursing in rural and remote communities means for nurses who have retired and continue to work, new opportunities can be created for older nurses to contribute to patient care and the health of their communities.

When nurses retire fully and leave rural and remote practice, their complex generalist nursing and community knowledge is lost. Acknowledging retired nurses’ resilience and strength while keeping them engaged in practice can benefit the nurses themselves, their younger colleagues, the community, and the workplace. When retired nurses have opportunities to mentor newer nurses, they can pass on clinical reasoning expertise to those just entering rural and remote practice and contribute their joy for living and working in rural and remote communities.

To sustain older rural and remote nurses, there needs to be a greater emphasis on professional and personal supports, in particular, place-based processes that are geared to RNs’ and LPNs’ life stage, professional practice, and type of practice. This study has shown that such work-focused supports are necessary but may not be sufficient to address the essential components of community engagement and fostering the practice of older nurses in rural and remote communities.

## Data Availability

The dataset generated and analyzed during the current study is not publicly available due to ethical commitments. For further information about the dataset contact the corresponding author (Dr. Martha MacLeod; martha.macleod@unbc.ca).

## Abbreviations

RN: Registered nurse
NP: Nurse practitioner
LPN: Licensed practical nurse (registered practical nurse in Ontario)
RPN: Registered psychiatric nurse
*RRNII*: Nursing Practice in Rural and Remote Canada study

## References

[1] Ross J, Mann S, Leonard G (2020). Rural nursing during the COVID-19 pandemic: a snapshot of nurses’ experiences from Aotearoa New Zealand. J Nurs Pract.

[2] Markowski M, Cleaver K, Weldon SM (2020). An integrative review of the factors influencing older nurses’ timing of retirement. J Adv Nurs.

[3] Buchan J, Catton H, Shaffer, FA. Ageing well? Policies to support older nurses at work. 2020. https://www.intlnursemigration.org/wp-content/uploads/2020/12/FINAL-Ageing-ICNM-Report-December-9-2020.pdf

[4] MacLeod MLP, Kulig JC, Stewart NJ. Lessons from 20 years of research on nursing practice in rural and remote Canada. Research in brief. Can Nurse. 2019; [May 13]. https://www.canadian-nurse.com/en/articles/issues/2019/may-2019/lessons-from-20-years-of-research-on-nursing-practice-in-rural-and-remote-canada.

[5] Ryan C, Bergin M, Wells JS (2017). Valuable yet vulnerable: a review of the challenges encountered by older nurses in the workplace. Int J Nurs Stud.

[6] Duffield C, Graham E, Donoghue J, Griffiths R, Bichel-Findlay J, Dimitrelis S. Why older nurses leave the workforce and the implications of them staying. J Clin Nurs. 2015;24(5–6):824–31. 10.1111/jocn.12747. 10.1111/jocn.12747 25524135

[7] Stewart NJ, MacLeod MLP, Kosteniuk JG, Olynick J, Penz KL, Karunanayake CP (2020). The importance of organizational commitment in predicting rural nurses’ intent to leave. J Adv Nurs.

[8] World Health Organization [WHO]. State of the world's nursing 2020: investing in education, jobs and leadership. Geneva: World Health Organization; 2020. License CC BY-NC-SA 3.0 IGO.S. https://www.who.int/publications/i/item/9789240003279.

[9] Canadian Institute for Health Information [CIHI] (2020). Nursing in Canada 2019: a lens on supply and workforce.

[10] Wakerman J, Humphreys J, Russell D, Guthridge S, Bourke L, Dunbar T (2019). Remote health workforce turnover and retention: what are the policy and practice priorities?. Hum Resour Health.

[11] Canadian Institute for Health Information (2019). Nursing in Canada 2018: a lens on supply and workforce.

[12] Australian Institute of Health and Welfare (2016). Nursing and midwifery workforce 2015.

[13] Smiley RA, Lauer P, Bienemy C, Berg JG, Shireman E, Reneau KA (2018). The 2017 National Nursing Workforce Survey. J Nurs Regul.

[14] Auerbach DI, Buerhaus PI, Staiger DO (2014). Registered nurses are delaying retirement, a shift that has contributed to recent growth in the nurse workforce. Health Aff.

[15] Canadian Institute for Health Information (2016). Regulated nurses 2015.

[16] Hewko S, Reay T, Estabrooks CA, Cummings GG (2019). The early retiree divests the health workforce: a quantitative analysis of early retirement among Canadian registered nurses and allied health professionals. Hum Resour Health.

[17] Uthaman T, Chua TL, Ang SY (2016). Older nurses: a literature review on challenges, factors in early retirement and workforce retention. Proc Singapore Healthc.

[18] Gan I (2020). A scoping review of the nursing workforce’s changing demography: supporting baby-boomer nurses. J Nurs Manag.

[19] Palumbo MV, McIntosh B, Rambur B, Naud S (2009). Retaining an aging nurse workforce: perceptions of human resource practices. Nurs Econ.

[20] Warburton J, Moor ML, Clune SJ, Hodgkin SP (2014). Extrinsic and intrinsic factors impacting on the retention of older rural healthcare workers in the north Victorian public sector: a qualitative study. Rural Remote Health.

[21] Voit K, Carson DB (2014). Post-retirement intentions of nurses and midwives living and working in the Northern Territory of Australia. Rural Remote Health.

[22] Cosgrave C, Malatzky C, Gillespie J (2019). Social determinants of rural health workforce retention: a scoping review. Int J Environ Res Public Health.

[23] Clendon J, Walker L (2016). Nurses aged over 50 and their perceptions of flexible working. J Nurs Manag.

[24] North N, Leung W, Lee R (2014). Aged over 50 years and practising: separation and changes in nursing practice among New Zealand’s older registered nurses. J Adv Nurs.

[25] Havens DS, Warshawsky NE, Vasey J (2013). RN work engagement in generational cohorts: the view from rural US hospitals. J Nurs Manag.

[26] Campbell CM, Patrician PA, Booth RZ (2020). Generational preferences in the nursing work environment: a dimensional concept analysis. J Nurs Manag.

[27] MacLeod MLP, Stewart NJ, Kosteniuk JG, Penz KL, Olynick J, Karunanayake CP (2019). Rural and remote registered nurses’ perceptions of working beyond their legislated scope of practice. Nurs Leaders (Tor Ont).

[28] MacLeod MLP, Stewart NJ, Kosteniuk JG, Penz KL, Olynick J, Karunanayake CP (2019). Rural and remote licensed practical nurses’ perceptions of working below their legislated scope of practice. Nurs Lead.

[29] MacLeod MLP, Stewart NJ, Kulig JC, Anguish P, Andrews M, Banner D (2017). Nurses who work in rural and remote communities in Canada: a national survey. Hum Resour Health.

[30] Gadamer HG (1960). Truth and method.

[31] Moules NJ, McCaffrey G, Field JC, Laing CM (2015). Conducting hermeneutic research: from philosophy to practice.

[32] Hsieh HF, Shannon SE (2005). Three approaches to qualitative content analysis. Qual Health Res.

[33] Moules NJ (2002). Hermeneutic inquiry: paying heed to history and Hermes - an ancestral, substantive, and methodological tale. Int J Qual Methods.

[34] du Plessis V, Beshiri R, Bollman RD, Clemenson H. Definitions of rural. Rural and Small Town Canada Analysis Bulletin, Stats Canada. 2001;3(3)*.* https://www150.statcan.gc.ca/n1/en/catalogue/21-006-X

[35] Kulig JC, Andrews ME, Stewart NJ, Pitblado JR, MacLeod MLP, Bentham D (2008). How do registered nurses define rurality?. Aust J Rural Health.

[36] Stewart NJ, D’Arcy C, Pitblado JR, Morgan DG, Forbes D, Remus G (2005). A profile of registered nurses in rural and remote Canada. Can J Nurs Res.

[37] Dillman D, Smyth JD, Christian LM (2014). Internet, phone, mail, and mixed-mode surveys: the tailored design method.

[38] Walker L, Clendon J, Willis J (2018). Why older nurses leave the profession. Kai Tiaki Nurs Res.

[39] Fisher GG, Chaffee DS, Sonnega A (2016). Retirement timing: a review and recommendations for future research. Work Aging Retire.

[40] Stanley D, Stanley K (2019). Clinical leadership and rural and remote practice: a qualitative study. J Nurs Manag.

[41] Fragar LJ, Depczynski JC (2011). Beyond 50. Challenges at work for older nurses and allied health workers in rural Australia: a thematic analysis of focus group discussions. BMC Health Serv Res.

[42] Day G, Robert G, Rafferty AM (2020). Gratitude in health care: a meta-narrative review. Qual Health Res.

[43] Harris K, Krygsman S, Waschenko J, Rudman DL (2018). Ageism and the older worker: a scoping review. Gerontologist.

[44] Kwok C, Bates KA, Ng ES (2016). Managing and sustaining an ageing nursing workforce: identifying opportunities and best practices within collective agreements in Canada. J Nurs Manag.

[45] Bordia P, Read S, Bordia S (2020). Retiring: role identity processes in retirement transition. J Organ Behav.

[46] Beehr TA, Bennett MM (2015). Working after retirement: features of bridge employment and research directions. Work Aging Retire.

